# Hormone-Dependent Cancers: Molecular Mechanisms and Therapeutical Implications

**DOI:** 10.3390/cells12010110

**Published:** 2022-12-28

**Authors:** Günter Emons

**Affiliations:** Department of Gynecolgy and Obstetrics, University Medicine Göttingen, Robert Koch Str. 40, 37075 Göttingen, Germany; emons@med.uni-goettingen.de; Tel.: +49-551-3795687

Hormone-dependent cancers of the breast and prostate are the most common cancers in women and men, respectively. In addition, cancers of the ovaries and the endometrium are frequent and hormone-dependent. Endogenous and exogeneous steroids as well as proteo- and peptide hormones play essential roles in the development and progression of these hormone-dependent malignancies. Pharmacological manipulations of these endocrine mechanisms are a cornerstone of the treatment of these tumors, which eventually develop resistance to endocrine therapy.

In this Special Issue of *Cells*, five up-to-date reviews address important aspects of the current knowledge of hormone action in breast, prostate, ovarian, and endometrial cancers [1,2,3,4,5]. Three original studies are devoted to the role of human chorionic gonadotropin in breast cancer [6], the regulation of the invasion of breast cancer cells [7], and the role of cholesterol as an endogenous ligand of estrogen-related receptor alpha [8].

Hormone-receptor-positive breast cancers account for 75% of new breast cancer diagnoses. Primary or acquired resistance to endocrine therapies represents a clinically relevant issue and is largely responsible for disease recurrence after primary therapy and cancer progression in the metastatic setting. Zattarin and co-authors give a comprehensive review of the molecular mechanisms and clinical implications of the loss of estrogen/progesterone receptors, which is important in primary or acquired resistance of breast cancer to endocrine therapy. In particular, the effect of neoadjuvant endocrine therapies is analyzed [1].

A high-risk type of breast cancer is the so-called “triple-negative breast cancer” (TNBC), lacking the expression of estrogen receptor alpha (ER-alpha) and progesterone receptors (PRs) and showing no overexpression of human epidermal growth factor receptor 2 (HER-2). This breast cancer is considered to be not amenable to therapies targeted to ER-alpha, PRs, or HER-2. Treeck et. al review the notion that estrogen signaling in TNBCs can be well-activated through estrogen receptor-beta, G-protein-coupled estrogen receptor 1 (GPER-1), and constitutively active estrogen-related receptors (ERRs), and they discuss options for endocrine therapy strategies in TNBCs [2].

Human chorionic gonadotropin (hCG) binds specifically to luteinizing hormone receptors. Dando and colleagues provide data that suggest this hormone stimulates the proliferation of MCF-7 breast cancer cells and the differentiation of MCF-7 stem cells [6].

Kolb and coworkers analyzed some of the mechanisms mediating invasiveness and aggressiveness of mesenchymally transformed breast cancer cells [7].

An increased risk of breast cancer has been associated with high dietary cholesterol intake. Ghanbari et al. show that cholesterol acts as an endogenous ligand for estrogen-related receptor alpha (ERRalpha) and stimulates various mechanisms in estrogen-receptor-positive and triple-negative breast cancers [8].

Most prostate cancers (PCs) are androgen-dependent in their early stage, and androgen deprivation (ADT) represents the standard treatment. However, after a few years, most patients progress toward the so called “castration-resistant prostate cancer”, characterized by tumor growth even in the presence of castration levels of circulating androgens. Fontana and Limonta present a comprehensive review of the molecular mechanisms underlying the development of this resistance and options to overcome it, including direct effects of gonadotropin-releasing hormone (GnRH) and its analogues on resistant tumor cells [3].

Ovarian cancer is the fifth most common cancer-associated cause of death in women. Gonadotropin-releasing hormone (GnRH) stimulates the pituitary secretion of luteinizing hormone (LH) and follicle-stimulating hormone (FSH). These gonadotropins might stimulate the proliferation of ovarian cancer cells. The suppression of pituitary gonadotropin secretion by superactive GnRH-analogues might have a therapeutical effect. In addition, a local GnRH/GnRH receptor system has been elucidated in ovarian cancer. Gründker and Emons review the present knowledge on GnRH and its analogues, as well as possible therapeutical applications in ovarian cancer [4].

Endometrial cancer (EC) is one of the most common cancers in women. Although the endometrium is a classical target organ for estrogens and progestins, the therapeutical efficacy of endocrine therapies is limited. Emons and Gründker review the extensive literature on the expression and molecular mechanisms of GnRH systems in EC and the clinical data available on the treatment of advanced EC with various analogues of GnRH [5].

This Special Issue of *Cells* provides comprehensive reviews of important aspects of hormone-dependent cancers and some of the most interesting findings on possible new approaches.
